# Septic and Crystal-Induced Arthritis (Pseudogout) Post-COVID-19 Vaccination

**DOI:** 10.7759/cureus.23902

**Published:** 2022-04-06

**Authors:** Felix W Wireko, Sabah Khalafalla, Tahereh Jamshidi, Siham Mahgoub

**Affiliations:** 1 Internal Medicine, Howard University Hospital, Washington, DC, USA; 2 Rheumatology, Howard University Hospital, Washington, DC, USA; 3 Infectious Disease, Howard University Hospital, Washington, DC, USA

**Keywords:** calcium pyrophosphate deposition disease (cppd), vaccine-associated complications, osteoarthritis, covid-19 vaccination, septic arthritis

## Abstract

Severe acute respiratory syndrome coronavirus 2 infection has been a global public health crisis for the past two years. Vaccination has been a mainstay preventive approach among other strategies such as hand washing, social distancing, and wearing facemasks. Here, we present a case of concomitant calcium pyrophosphate deposition disease flare and septic arthritis of the right knee following coronavirus disease 2019 (COVID-19) booster vaccination in a 69-year-old African American male who presented with a painful swollen right knee with associated fever, chills, and rigors three days post-vaccination. Right knee synovial fluid aspirate appeared turbid with elevated white cell count, positive for both intra and extracellular calcium pyrophosphate crystals, and positive for beta-hemolytic *Streptococcus* group C. The swollen joint improved with right knee arthroscopic irrigation and intravenous antibiotics on admission. The patient subsequently completed a total of six weeks of antibiotics with clinical improvement and normalization of inflammatory markers. No reported incidence of gout or pseudogout post-COVID-19 vaccination has been reported despite reported cases of gout flares with other vaccines. Improper aseptic vaccination technique has been implicated as a possible cause of septic arthritis post-vaccination. Healthcare providers must discuss such adverse events with their patients prior to vaccine administration.

## Introduction

Coronavirus disease 2019 (COVID-19) caused by the severe acute respiratory syndrome coronavirus 2 (SARS-CoV-2) was declared a public health emergency soon after it first emerged in Wuhan, China, in December 2019 [1]. The disease was subsequently declared a pandemic by the World Health Organization on March 11, 2020 [2]. Since then, the search for treatment and prevention has been rapidly evolving. Hand washing, social distancing, facial mask, and quarantining were among the precautions taken to limit the spread of COVID-19 [3]. Vaccination is one of the long-proven approaches to preventing infectious diseases. Several types of COVID-19 vaccines have been introduced and approved for use globally. In the United States, the Food and Drug Administration granted the Emergency Use Authorization to both COVID-19 mRNA vaccines (BNT162b2 Pfizer-BioNTech and Moderna) and the adenoviral vector vaccine Ad26.COV2.S (Johnson & Johnson).

Since the COVID-19 vaccine rollout, varying degrees of side effects have been reported, ranging from common fatigue, injection site pain, headache, myalgia, and chills to more severe effects, including thrombosis and myocardial infarction [4,5]. Here, we present a case of concomitant calcium pyrophosphate deposition disease (CPPD) flare and septic arthritis of the right knee following COVID-19 booster vaccination.

## Case presentation

**Figure 1:**
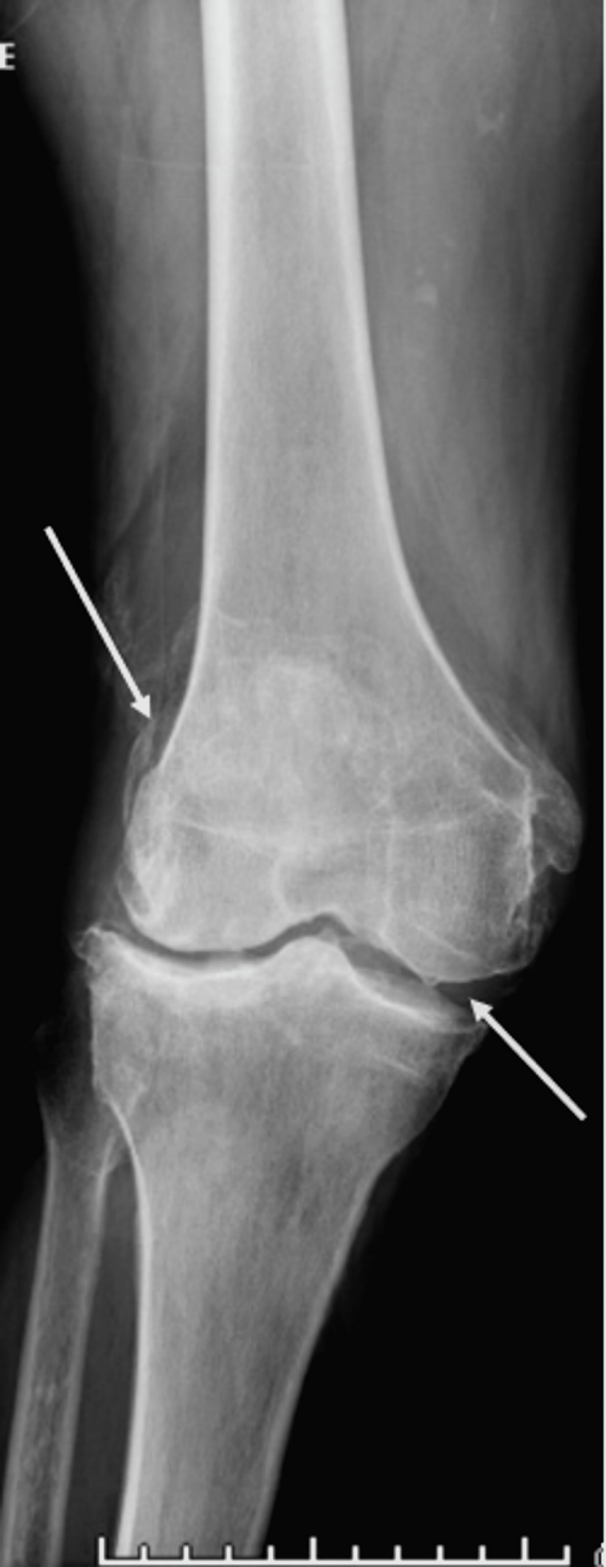
X-ray of the right knee showing osteoarthritis with suprapatellar joint effusion and evidence of chondrocalcinosis.

**Figure 2:**
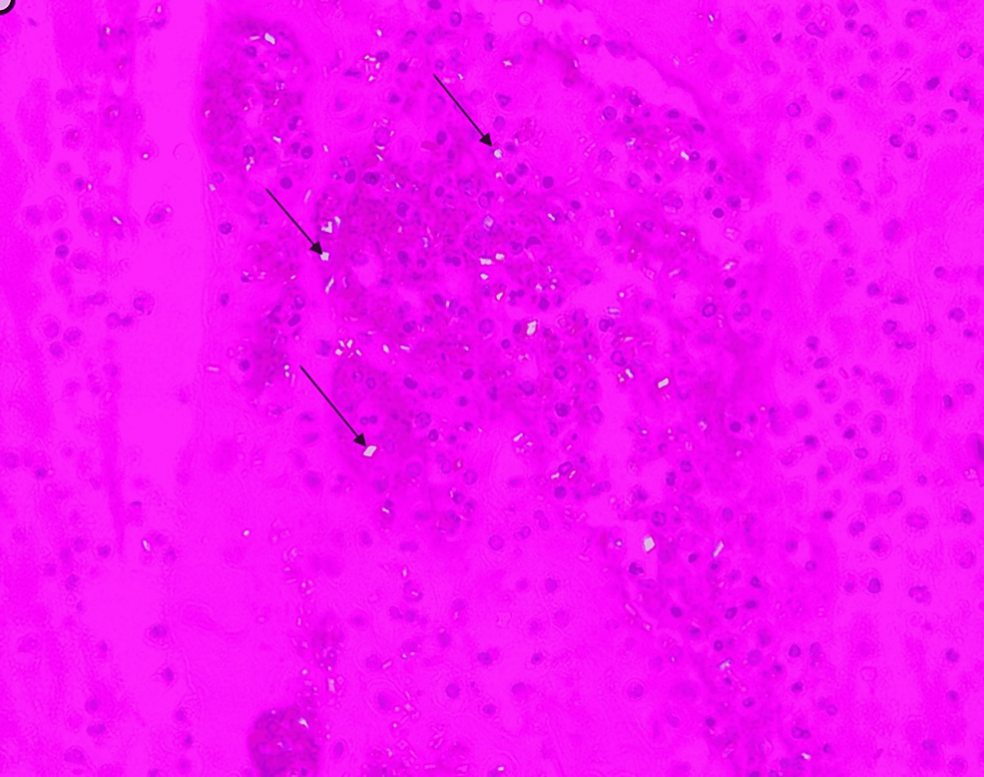
Synovial fluid of the right knee showing intra and extracellular rhomboid-shaped CPP crystals. CPP: calcium pyrophosphate

A 69-year-old African American male with hypertension and right knee osteoarthritis history presented to our hospital with a painful swollen right knee three days after receiving his COVID-19 vaccine booster (Pfizer) on the right deltoid region. Six months earlier, he had received two doses of the Pfizer COVID-19 vaccine with no side effects. However, a day after his booster vaccine, he developed fever, chills, rigors, and body aches. He also experienced right knee pain with no antecedent trauma. The patient was seen in a different health facility and discharged for follow-up with orthopedic surgery. Due to worsening of his right knee pain and swelling, he sought care in our hospital. Upon presentation, he was afebrile (97.6°F), with stable vital signs. The musculoskeletal examination was notable for right knee effusion, tenderness, and reduced range of motion. Laboratory tests showed leukocytosis with a white blood cell (WBC) count of 13 × 10^9^ and elevated inflammatory markers, C-reactive protein of 59.6 mg/dL, and erythrocyte sedimentation rate of 90 mm/hour. SARS-CoV-2 RNA was negative. His right knee X-ray revealed moderate tri-compartmental osteoarthritis with suprapatellar joint effusion and evidence of faint chondrocalcinosis (Figure 1). Right knee synovial fluid aspirate appeared turbid with high a WBC (105,080 mm^9^) with 66% polymorphs, positive for intra and extracellular calcium pyrophosphate crystals (Figure 2) and gram-positive cocci in chains. The patient underwent right knee arthroscopic irrigation, which revealed purulent effusion in the right knee joint. The right knee aspirate culture revealed beta-hemolytic *Streptococcus* group C. The patient received a seven-day course of 2 g ceftriaxone during hospitalization. He completed six weeks of antibiotics with clinical improvement and normalization of inflammatory markers.

## Discussion

Since the introduction of the COVID-19 vaccine, multiple side effects have been reported, ranging from injection site reactions, fever, malaise, and fatigue to more severe conditions such as pericarditis, myocarditis, thrombotic thrombocytopenic purpura, and anaphylaxis; however, the benefits far outweigh the risks [5]. More recently, case reports of arthritis, including septic arthritis, have been reported in relation to the COVID-19 vaccine [6,7]. Immune-mediated diseases can also flare up following COVID-19 vaccination but are rare and responsive to therapy [8]. On searching the published literature in English and applying filters on PubMed and Google Scholar, as of February 2022, using the search terms of COVID-19 vaccination, pseudogout, and gout, we found no reported incidence of gout or pseudogout with COVID-19 vaccination. In contrast, there have been cases of gout flares with other vaccines, such as the recombinant zoster vaccine. This reaction is speculated to be mediated by the NLR family pyrin domain containing 3 (NLRP3) inflammasome, activated by aluminum adjuvants contained in most routine adult vaccines [9]. The COVID-19 vaccine (Pfizer-BioNTech) does not contain aluminum adjuvants [10], and, thus, a different mechanism from the broader range of stimuli activation may be involved. Acute illnesses, such as infections, are major risk factors for the flares of gout and pseudogout, which are seen in most inpatient admissions [11]. Risk factors for septic arthritis include advanced age, skin and soft tissue infection, intravenous drug use, pre-existing joint disease, and immunosuppression [12]. The acute septic arthritis in this patient could have triggered the pseudogout flare, as evident in the synovial fluid analysis with extra and intracellular CPP deposits. Moreover, this patient had a pre-existing joint disease and a recent soft tissue injection (COVID-19 vaccination), which might have played a role in the pathogenesis. Although less likely, it is possible that aseptic techniques were not used during the patient’s vaccination and possibly resulted in septic arthritis and not necessarily provoked by the vaccine.

## Conclusions

Flares of pseudogout and gout after COVID-19 vaccination are uncommon. However, with our case report, we would like to highlight the possibility of similar adverse events and alert healthcare providers to discuss such adverse events with their patients before vaccine administration, especially in susceptible and predisposed individuals. It is also important that existing institutional guidelines and mechanisms to report potential adverse effects post-vaccination are followed to capture all such events to ensure patients’ safety.
